# Supplementary material for: Oxidative stress changes interactions between 2 bacterial species from competitive to facilitative
Source: PLoS Biol. 2024 Feb 5;22(2):e3002482. doi: 10.1371/journal.pbio.3002482 (PMC10881020; doi:10.1371/journal.pbio.3002482)
Supplement: S1 Appendix — Descriptions of models 1 with implicit toxicity and model 2 with explicit toxicity due to ROS. All code is available at https://zenodo.org/records/10396269. (PDF) [file pbio.3002482.s001.pdf]

S1 Appendix: Details on the modeling and fitting analysis.

Model with implicit toxicity (Model 1)

We fit the data from the experiments to a mathematical model to predict whether species are expected to engage in competition, facilitation, and/or to coexist over long term serial transfers.

The first model is a modified Monod model with maximum growth rate r, half-saturation constant K, and yield Y, in which we incorporate a mortality term to take into account concentration-dependent toxicity of linoleic acid (LA). We assume that toxicity of the environment $T(t)$ increases linearly over time and is proportional to the LA concentration $T(t) = (\beta + \gamma t)$ (in a simpler version of this model, we assume that toxicity is constant, $\gamma = 0$, cf S6 Fig).

The equations that we start from are for a single species B in a batch culture with LA (C):

$$\frac{\mathrm{d}B}{\mathrm{d}t} = \left(\frac{r}{C(t)+K} - (\beta + \gamma t)\right) C(t)B(t) \quad (6)$$

$$\frac{\mathrm{d}C}{\mathrm{d}t} = -\frac{1}{Y}\frac{r}{C(t)+K}C(t)B(t) \quad (7)$$

The equations for two species (B_1, corresponding to Ct and B_2, corresponding to At) in co-culture in LA are:

$$\frac{\mathrm{d}B_1}{\mathrm{d}t} = \left(\frac{r_1}{C(t)+K_1} - (\beta_1 + \gamma_1 t)\right) C(t)B_1(t) \quad (8)$$

$$\frac{\mathrm{d}B_2}{\mathrm{d}t} = \left(\frac{r_2}{C(t)+K_2} - (\beta_2 + \gamma_2 t)\right) C(t)B_2(t) \quad (9)$$

$$\frac{\mathrm{d}C}{\mathrm{d}t} = -\frac{1}{Y_1}\frac{r_1}{C(t)+K_1}C(t)B_1(t) - \frac{1}{Y_2}\frac{r_2}{C(t)+K_2}C(t)B_2(t) \quad (10)$$

Because the bacteria show some growth in the minimal medium, we assume an additional unknown nutrient to be present in the minimal medium, whose concentration $N(t)$ is modelled in an additional equation. The updated model becomes:

$$\frac{\mathrm{d}B_1}{\mathrm{d}t} = \left(\frac{r_{C1}}{C(t)+K_{C1}} - (\beta_1 + \gamma_1 t)\right) C(t)B_1(t) + \frac{r_{N1}}{N(t)+K_{N1}}N(t)B_1(t) \quad (11)$$

$$\frac{\mathrm{d}B_2}{\mathrm{d}t} = \left(\frac{r_{C2}}{C(t)+K_{C2}} - (\beta_2 + \gamma_2 t)\right) C(t)B_2(t) + \frac{r_{N2}}{N(t)+K_{N2}}N(t)B_2(t) \quad (12)$$

$$\frac{\mathrm{d}C}{\mathrm{d}t} = -\frac{1}{Y_{C1}}\frac{r_{C1}}{C(t)+K_{C1}}C(t)B_1(t) - \frac{1}{Y_{C2}}\frac{r_{C2}}{C(t)+K_{C2}}C(t)B_2(t) \quad (13)$$

$$\frac{\mathrm{d}N}{\mathrm{d}t} = -\frac{1}{Y_{N1}}\frac{r_N 1}{C(t)+K_{N1}}C(t)B_1(t) - \frac{1}{Y_{N2}}\frac{r_{N2}}{C(t)+K_{N2}}C(t)B_2(t) \quad (14)$$

We then first estimate the parameters of the growth in the minimal medium using an arbitrary concentration for this unknown nutrient (0.01), by using the data from both mono- and co-cultures of Ct (species B_1) and At (species B_2) in the minimal medium (S8 Fig). This allows us to obtain estimates for the parameters r_{N1}, r_{N2}, Y_{N1}, Y_{N2}, K_{N1} and K_{N1}. Then, we fix these parameters and estimate the parameters for the growth of At and Ct in mono-culture using a range of concentrations of LA (0.05%, 0.1%, 0.5% and 0.075%). This allows us to estimate r_{C1}, r_{C2}, Y_{C1}, Y_{C2}, K_{C1}, K_{C1} and the toxicity parameters for Ct (β_1 and γ_1) and At (β_2 and γ_2). .

Model with explicit toxicity due to ROS (Model 2)

In this second model, we add a new state variable corresponding to the concentration of ROS. LA is now only a nutrient, and the toxicity is proportional to ROS concentration (which can increase), so we do not need a specific parameter for the toxicity accumulation. The parameters β_1 and β_2 are the sensitivity of Ct and At to ROS (high value meaning low tolerance). The uptake of LA does not change from the previous model. The ROS intrinsic dynamics depend on their production by the oxidation of LA (spontaneous oxidation at rate d, positive feedback by ROS presence in the media, e, their half-life l and the yield of ROS production m; in a simpler version of this model, we assume no positive feedback, $e = 0$, cf S9 Fig). To this intrinsic part, we add the detoxification by the cells, through parameters α_1 for Ct and α_2 for At.

The co-culture equations become:

$$\frac{\mathrm{d}B_1}{\mathrm{d}t} = \frac{r_{C1}}{C(t)+K_{C1}}C(t)B_1(t) - \beta_1 B_1(t)R(t) + \frac{r_{N1}}{N(t)+K_{N1}}N(t)B_1(t) \tag{15}$$

$$\frac{\mathrm{d}B_2}{\mathrm{d}t} = \frac{r_{C2}}{C(t)+K_{C2}}C(t)B_2(t) - \beta_2 B_2(t)R(t) + \frac{r_{N2}}{N(t)+K_{N2}}N(t)B_2(t) \tag{16}$$

$$\frac{\mathrm{d}C}{\mathrm{d}t} = -\frac{1}{Y_{C1}}\frac{r_{C1}}{C(t)+K_{C1}}C(t)B_1(t) - \frac{1}{Y_{C2}}\frac{r_{C2}}{C(t)+K_{C2}}C(t)B_2(t) - \frac{1}{m}(d+eR(t))C(t) \tag{17}$$

$$\frac{\mathrm{d}N}{\mathrm{d}t} = -\frac{1}{Y_{N1}}\frac{r_N 1}{C(t)+K_{N1}}C(t)B_1(t) - \frac{1}{Y_{N2}}\frac{r_{N2}}{C(t)+K_{N2}}C(t)B_2(t) \tag{18}$$

$$\frac{\mathrm{d}R}{\mathrm{d}t} = (d+eR(t))C(t) - lR(t) - \alpha_1 B_1(t)R(t) - \alpha_2 B_2(t)R(t) \tag{19}$$

The mono-culture equations can be derived by setting one of the two bacterial densities to zero. Because we now have data on the spontaneous oxidation of LA in cell-free media, we can first estimate the parameters d, e, m, and l using the ROS proxy at different LA concentrations. We then use these fixed parameters to estimate the parameters of growth, toxicity, and detoxification for single species in mono-culture. Then, predictions can be made, as in the previous model, for the co-culture dynamics, short- and long-term dynamics (serial transfers), and mimicking the addition of a ROS quencher to the media (setting initial ROS concentration to zero, as well as parameters d, e, and l, figure not shown).

The best-fit parameter estimates for ROS intrinsic dynamics in model 2 are listed in S2 Table.
