# Supplementary figures and images for: Oxidative stress changes interactions between 2 bacterial species from competitive to facilitative
Source: PLoS Biol. 2024 Feb 5;22(2):e3002482. doi: 10.1371/journal.pbio.3002482 (PMC10881020; doi:10.1371/journal.pbio.3002482)

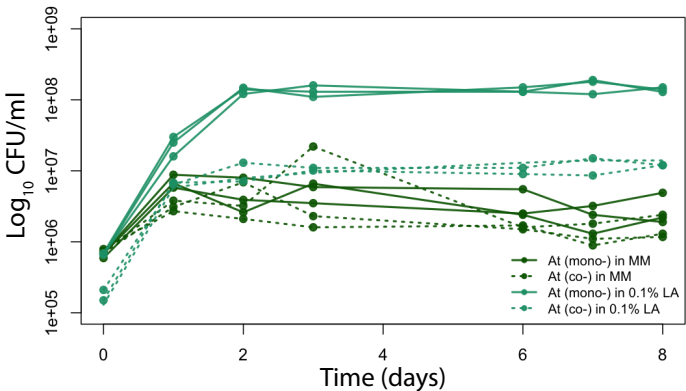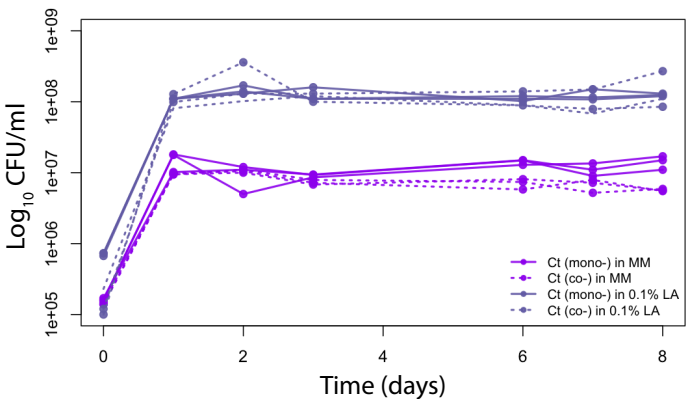

Supplement: S1 Fig — Growth in MM of At (top) and Ct (bottom) in the presence (dashed lines) and absence (solid lines) of the other species. We find that in all cases, bacteria grow in MM where no external carbon source has been added. Ct’s population size is only slightly inhibited by the presence of At in MM, while At grows similarly whether Ct is present or not. These data show that the competitive effect of Ct on At arises when 0.1% LA is added to MM, which is also shown on the plots. We argue then, that despite significant growth in MM, it is legitimate to focus on LA being the carbon source that is mediating the competitive effect. The data underlying this figure can be found at https://zenodo.org/records/8033845. (PDF) [file pbio.3002482.s002.pdf]

**A****Transfer simulation in 0.1% LA**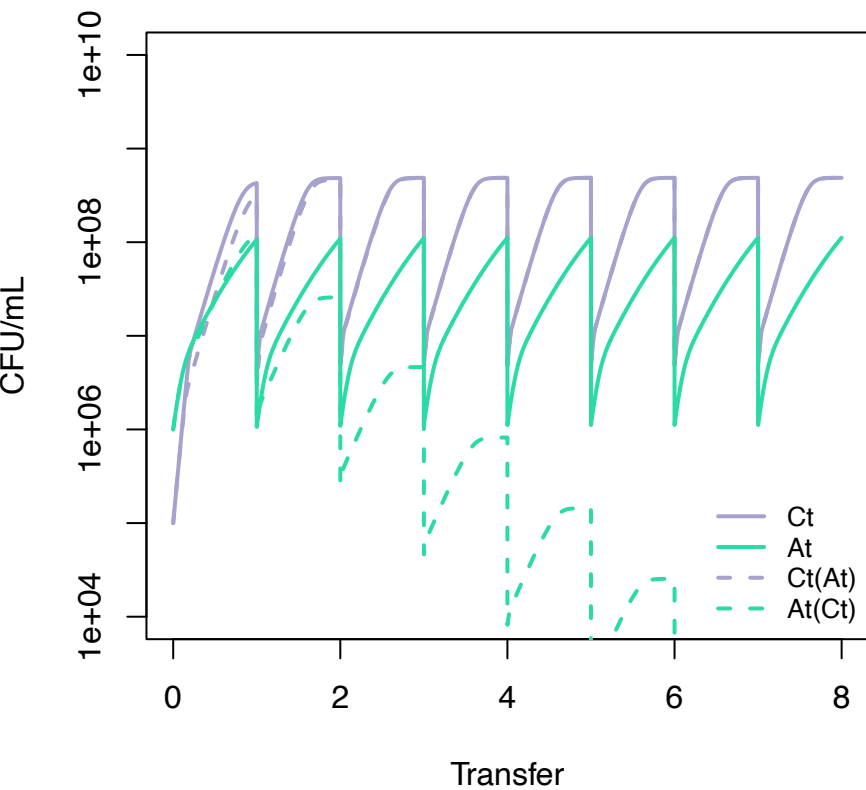**B****Transfer simulation in 0.75% LA**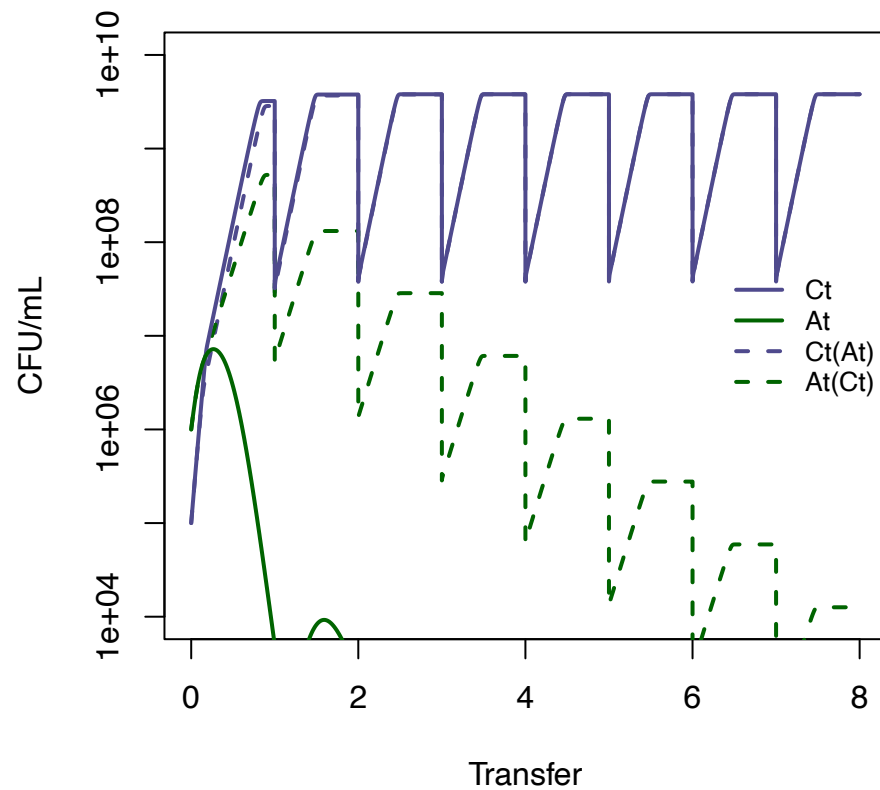

Supplement: S3 Fig — Transfer trajectories are simulated to predict the long-term coexistence of At and Ct according to the model. Here, we show for model 2, transfer of mono-cultures or co-cultures in the 2 LA conditions, with a dilution rate of 100. Even though after 5 transfers the 2 species are coexisting (above an extinction threshold), it is clear that this coexistence is not stable as At density is declining. Code that generated this figure is available at https://zenodo.org/records/10396269. (PDF) [file pbio.3002482.s004.pdf]

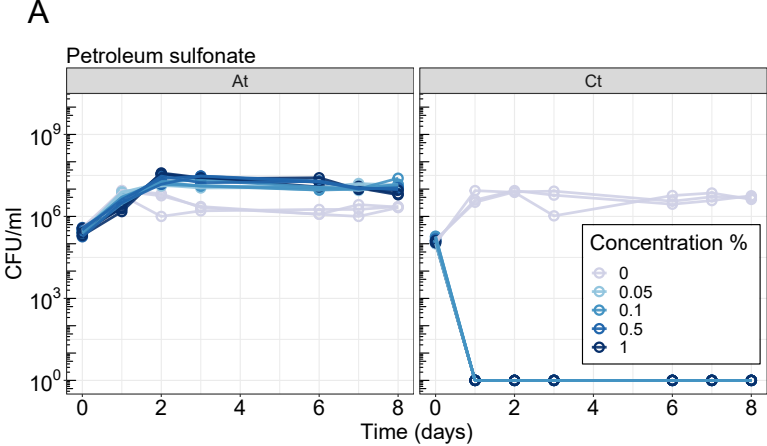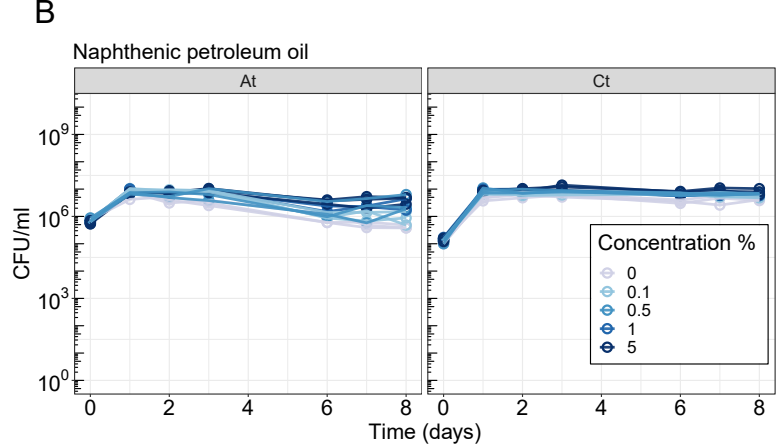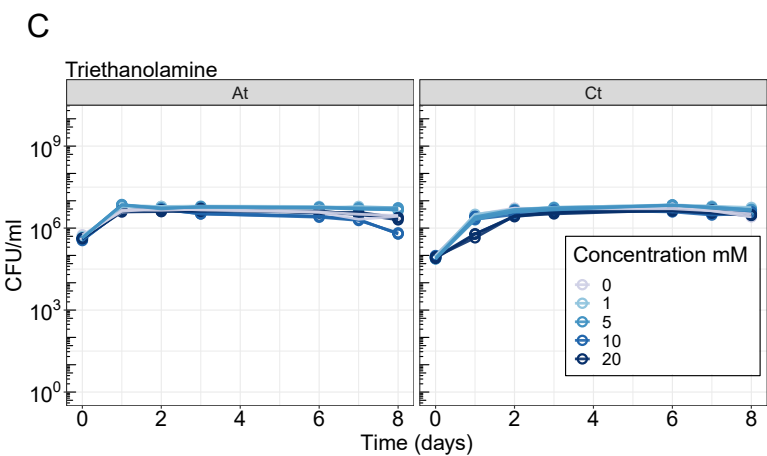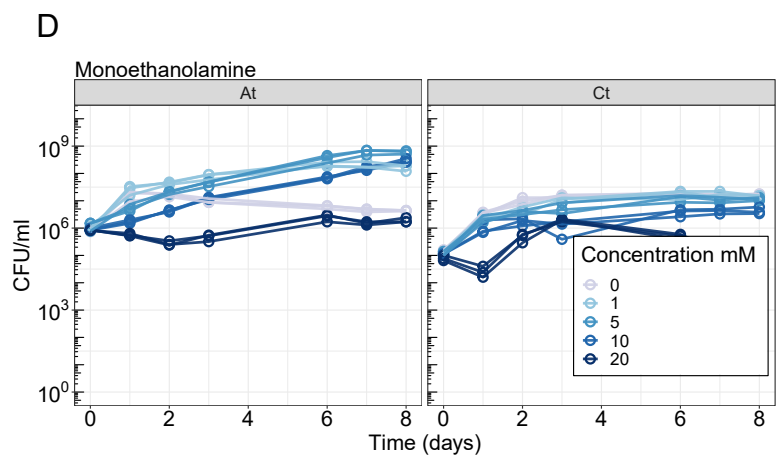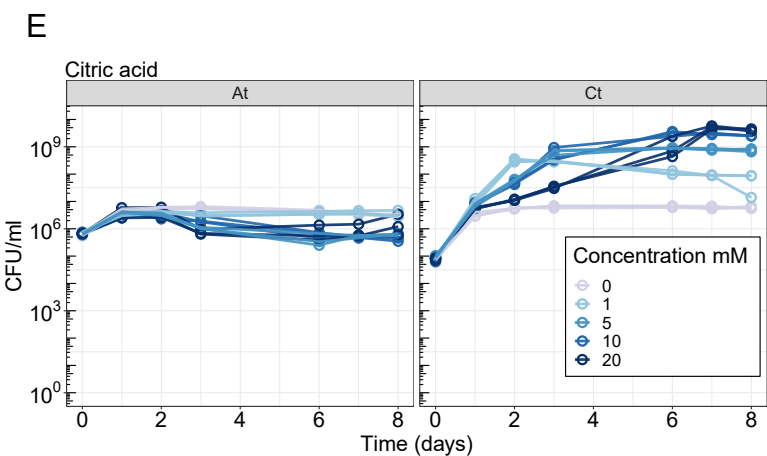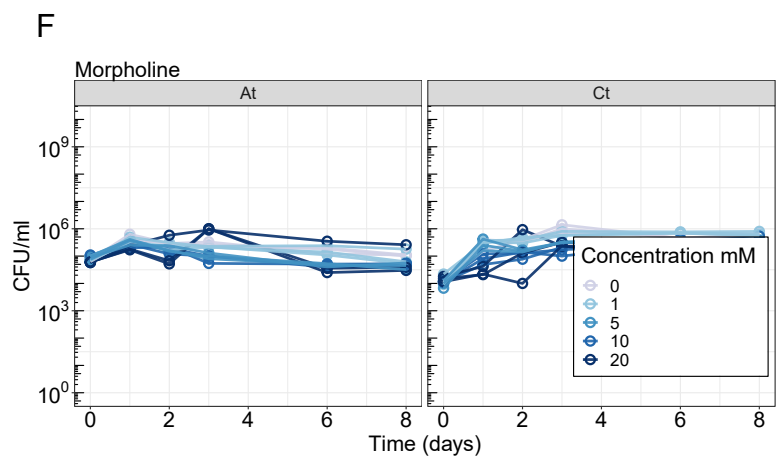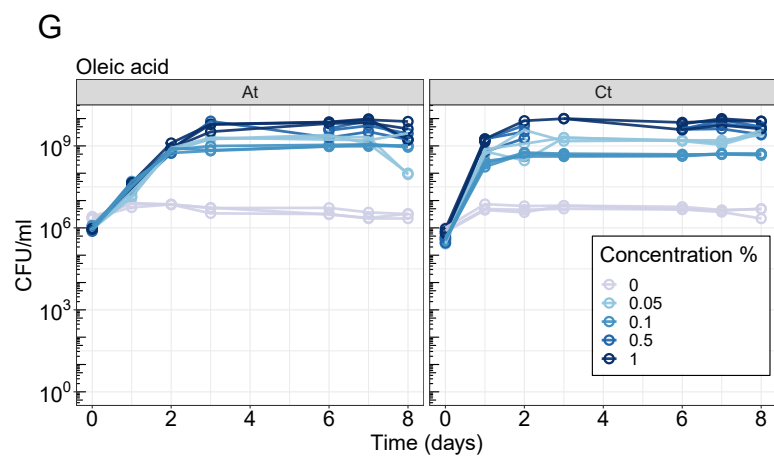

Supplement: S4 Fig — We chose compounds representative of MWF composition and grew At and Ct in increasing concentrations of the following compounds: petroleum sulfonate (A), naphthenic petroleum oil (B), triethanolamine (C), monoethanolamine (D), citric acid (E), morpholine (F), and oleic acid (G). Darker gradient of blue curves represents increasing compound concentration. (PDF) [file pbio.3002482.s005.pdf]

Adj R2 = 0.99253 Intercept = 0.0054018 Slope = 0.0018524 P = 4.2123e-25

Blank-subtracted absorbance at 532 nm

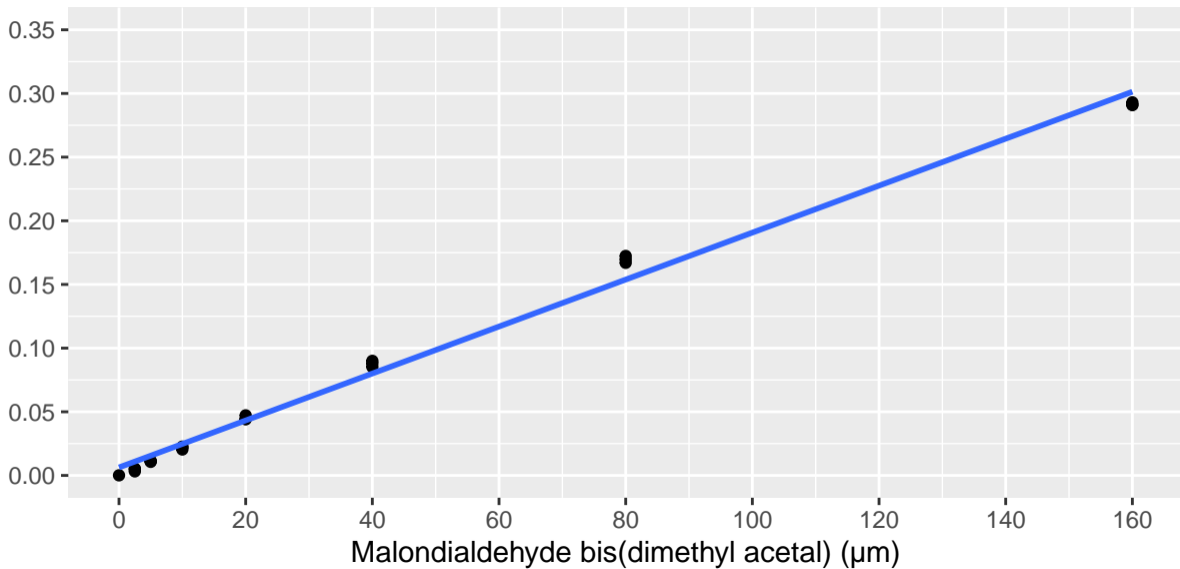

Supplement: S5 Fig — Malondialdehyde bis(dimethyl acetal) (MDA) concentration is used as a proxy for ROS accumulation: the higher the MDA concentration, the higher is the ROS abundance in the sample [45]. We chose 6 increasing concentrations of MDA (0, 2.5, 5, 10, 20, 40, 80, 160 μm) and we used 3 replicates per concentration to build a calibration curve. We performed the TBARS assay on each calibration sample as described in [45] and measured the 532 nm absorbance. We subtracted the absorbance of the blank (0 μm MDA) from each calibration sample and plotted MDA concentration versus blank-subtracted absorbance. We obtained a calibration curve and used its parameters to calculate the MDA concentration of the experimental samples shown in Fig 3. The data underlying this figure can be found at https://zenodo.org/records/8033845. (PDF) [file pbio.3002482.s006.pdf]

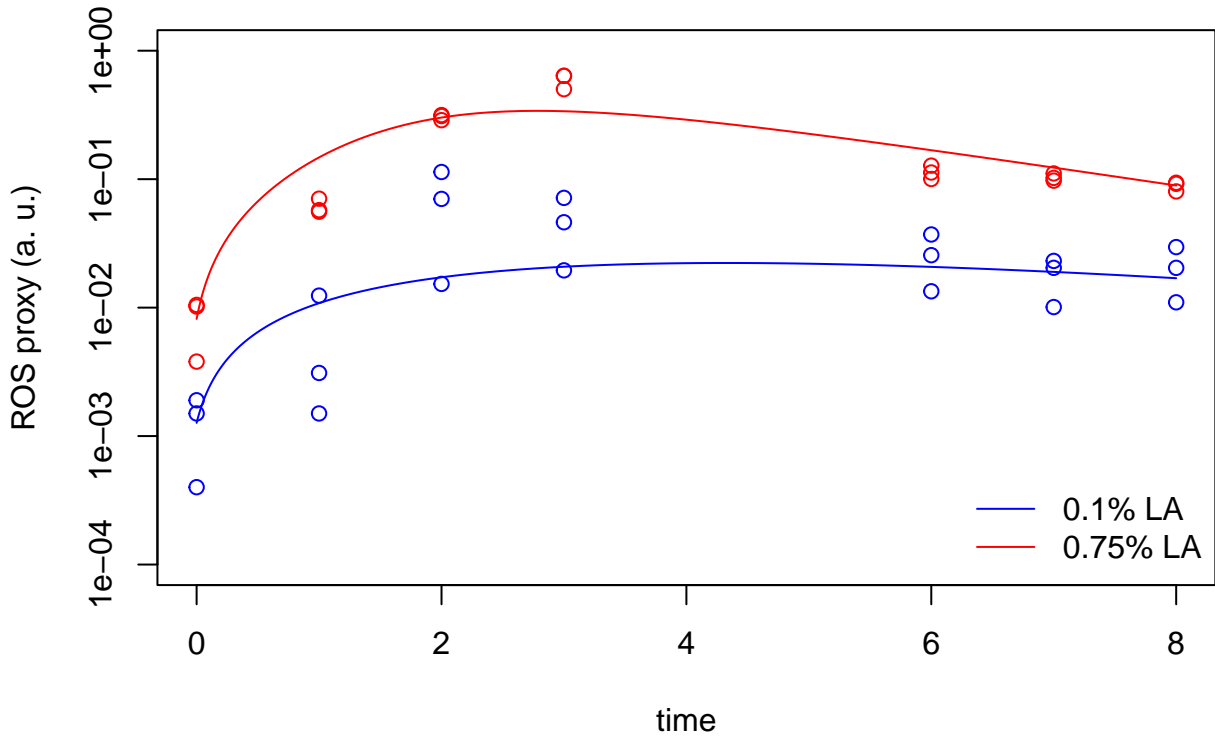

Supplement: S7 Fig — We used the MDA concentration in the cell-free media at both LA concentrations to estimate the intrinsic ROS parameters in the absence of bacteria. ROS concentration (in arbitrary units) first increases then stabilizes. The data underlying this figure can be found at https://zenodo.org/records/8033845 and code used for fitting is available at https://zenodo.org/records/10396269. (PDF) [file pbio.3002482.s008.pdf]

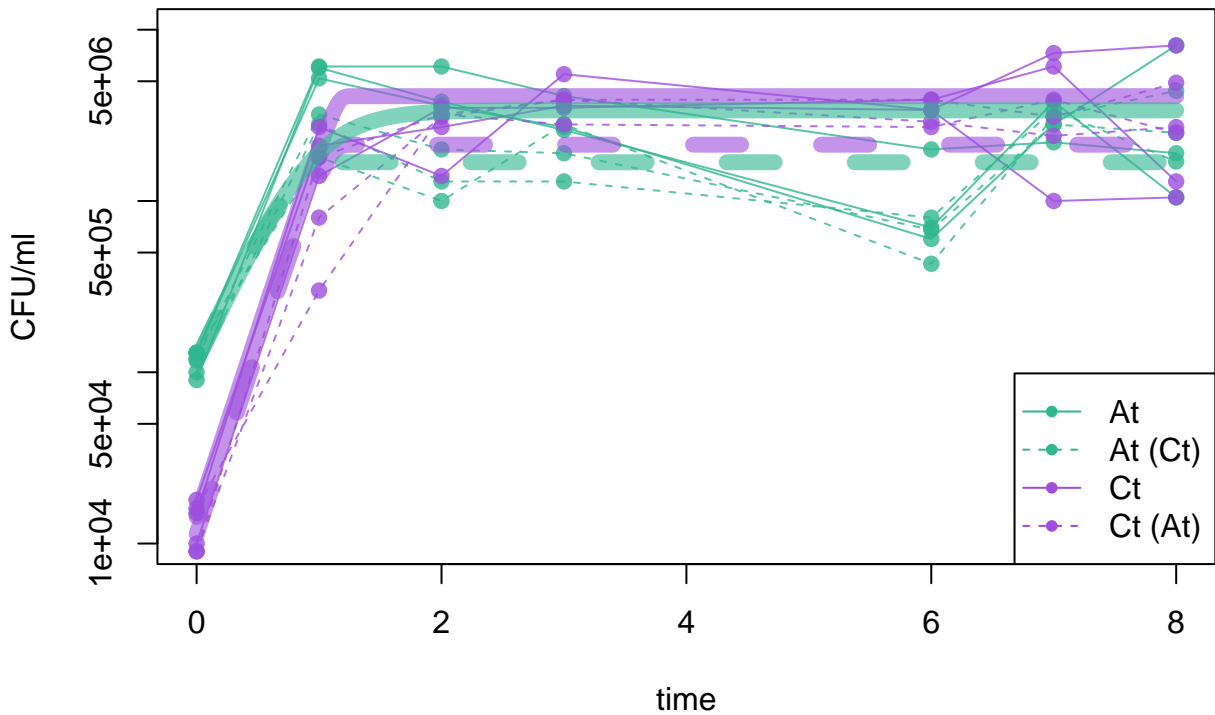

Supplement: S8 Fig — Since the nutrient that allows At and Ct to grow in the MM is unknown, we set it to an arbitrary concentration of 0.01 to fit the parameters of a consumer–resource model using both mono- and co-cultures of At and Ct. Here, the data used to fit the model are from the second experiment with ROS measurements. The model prediction is shown in the thick transparent lines, with solid lines for mono-cultures and dashed lines for co-cultures. The data underlying this figure can be found at https://zenodo.org/records/8033845 and code used for fitting is available at https://zenodo.org/records/10396269. (PDF) [file pbio.3002482.s009.pdf]

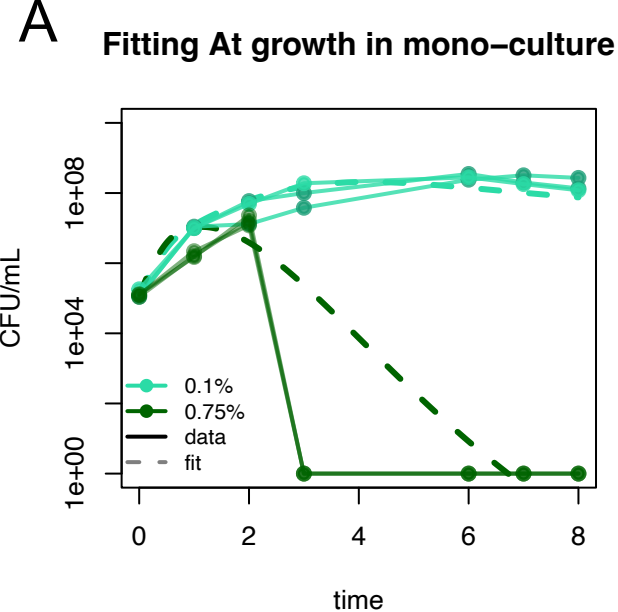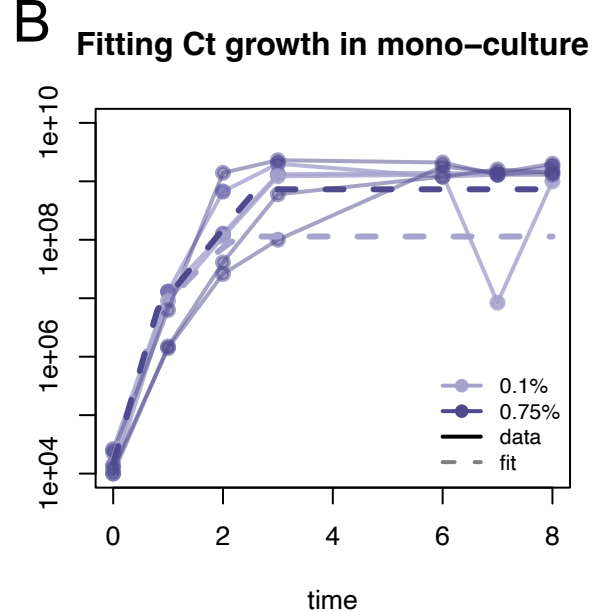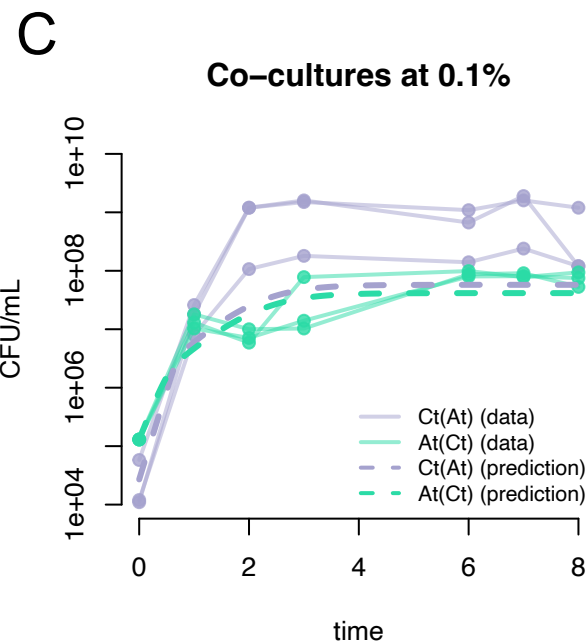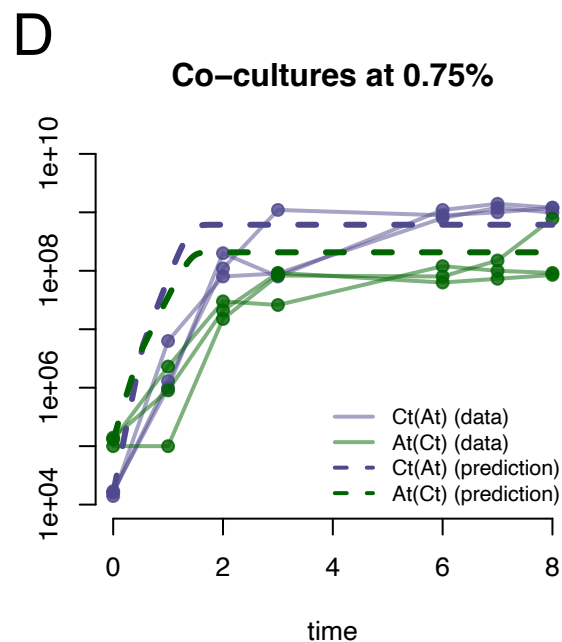

Supplement: S9 Fig — We consider a simpler model in which there is no ROS-induced oxidation of LA which removes the nontrivial feedback loop (e = 0 in Eqs 4 and 5). This model is quite similar to model 2 with the ROS-induced oxidation term but has a higher error both in the mono-culture fit and in terms of prediction of the co-culture growth (see S3 Table). Raw data are available at https://zenodo.org/records/8033845 and code used for fitting is available at https://zenodo.org/records/10396269. (PDF) [file pbio.3002482.s010.pdf]
