# Supplementary material for: Oxidative stress changes interactions between 2 bacterial species from competitive to facilitative
Source: PLoS Biol. 2024 Feb 5;22(2):e3002482. doi: 10.1371/journal.pbio.3002482 (PMC10881020; doi:10.1371/journal.pbio.3002482)
Supplement: S2 Fig — We tested the effect of 4 different concentrations of TBHQ (150 μm, 15 μm, 1.5 μm, and 0.375 μm) on each of the species in mono- and co-culture (A–D) and compared it to the MM alone (no TBHQ). We also tested the effect of DMSO, the solvent we used to prepare TBHQ, on growth (F). Based on this, we used MM + 1.5 μm as our antioxidant. The data underlying this figure can be found at https://zenodo.org/records/8033845. (PDF) [file pbio.3002482.s003.pdf]

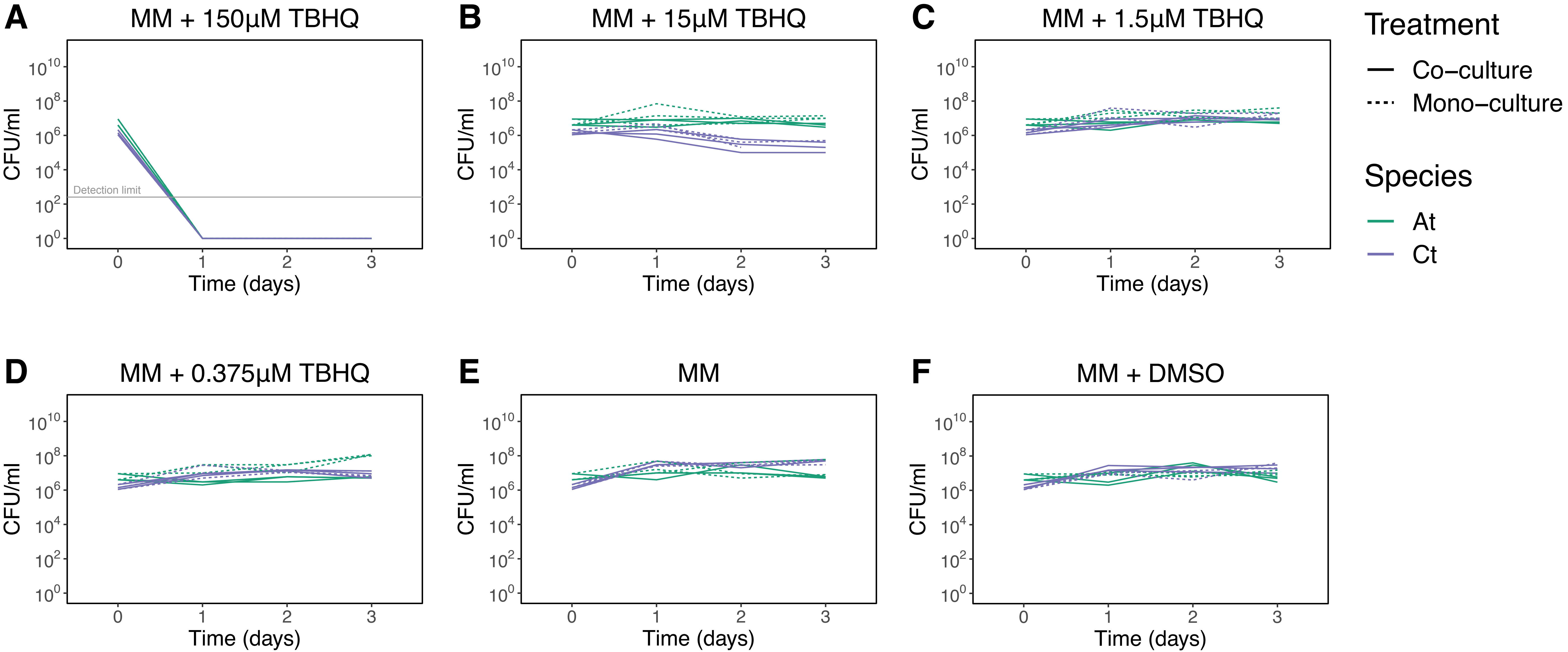
A
MM + 150µM TBHQ
B
MM + 15µM TBHQ
C
MM + 1.5µM TBHQ
D
MM + 0.375µM TBHQ
E
MM
F
MM + DMSO
CFU/ml
Time (days)
Detection limit
Treatment
Co−culture
Mono−culture
Species
At
Ct
