# Supplementary material for: Oxidative stress changes interactions between 2 bacterial species from competitive to facilitative
Source: PLoS Biol. 2024 Feb 5;22(2):e3002482. doi: 10.1371/journal.pbio.3002482 (PMC10881020; doi:10.1371/journal.pbio.3002482)
Supplement: S6 Fig — We consider a simpler model in which there is no toxicity accumulation (γ = 0 in Eq 1). LA is then directly toxic for At and the initial growth results from the consumption of the nutrients in the minimal medium. This model also captures the dynamics in mono-culture (panels A and B) and gives a qualitatively similar prediction for the co-cultures (panels C and D) as in the model with toxicity accumulation, but the error is larger because the growth of At is even more underestimated compared to the data (see S3 Table). The data underlying this figure can be found at https://zenodo.org/records/8033845 and code used for fitting is available at https://zenodo.org/records/10396269. (PDF) [file pbio.3002482.s007.pdf]

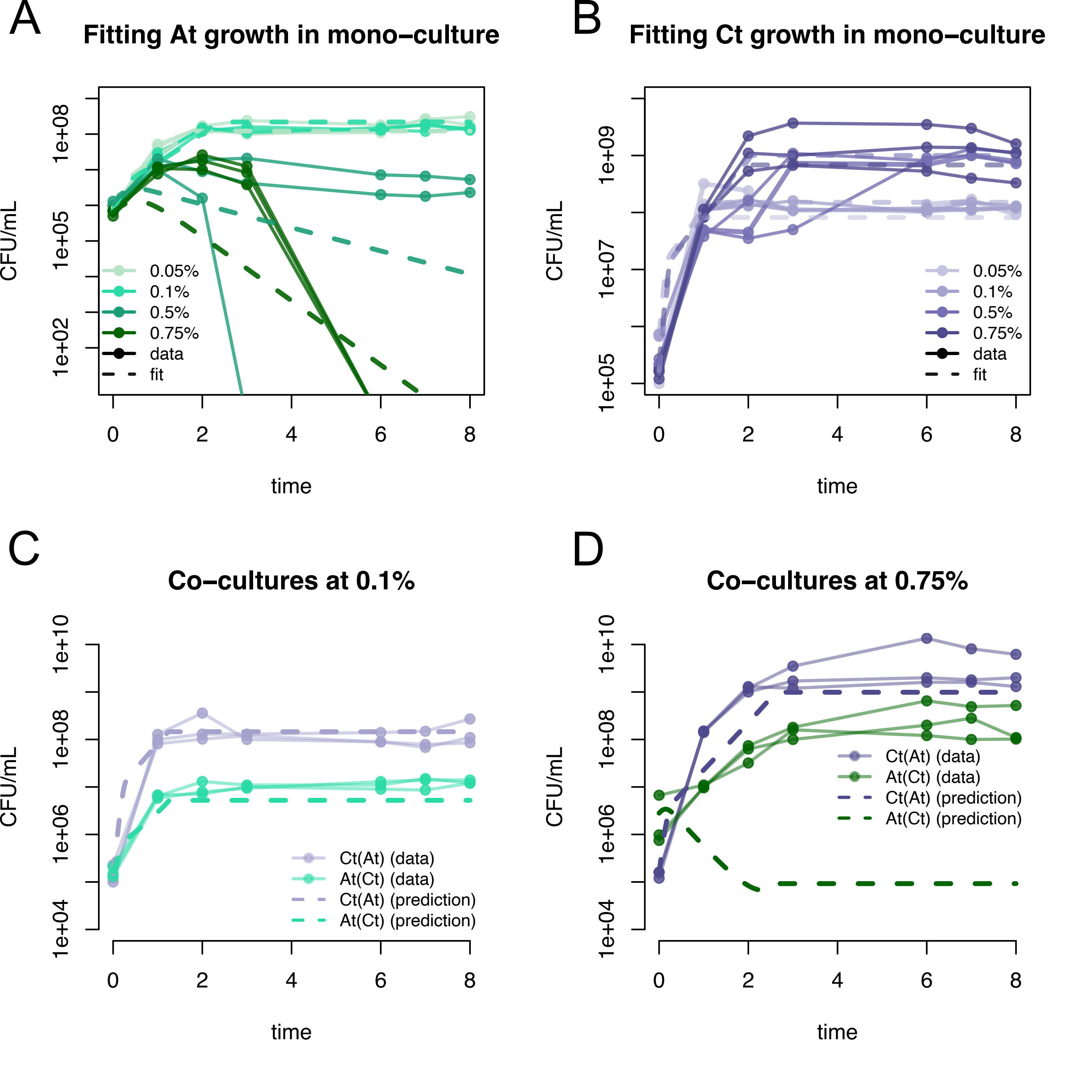
A
Fitting At growth in mono−culture
B
Fitting Ct growth in mono−culture
CFU/mL
1e+08
1e+05
1e+02
0.05%
0.1%
0.5%
0.75%
data
fit
1e+09
1e+07
1e+05
0
2
4
6
8
time
C
Co−cultures at 0.1%
D
Co−cultures at 0.75%
1e+10
1e+08
1e+06
1e+04
Ct(At) (data)
At(Ct) (data)
Ct(At) (prediction)
At(Ct) (prediction)
