# Supplementary material for: Oxidative stress changes interactions between 2 bacterial species from competitive to facilitative
Source: PLoS Biol. 2024 Feb 5;22(2):e3002482. doi: 10.1371/journal.pbio.3002482 (PMC10881020; doi:10.1371/journal.pbio.3002482)
Supplement: S1 Table — We searched for a list of putative ROS-degrading enzymes from a recent review paper [54] by searching the annotated genes of our 2 strains, based on sequences available on NCBI (Accession: PRJNA991498). We show the gene families listed in [54] and whether we found genes of the same family in our 2 genomes with gene number shown in brackets. This analysis shows that gene presence/absence tells us little about which of the 2 strains is more resistant to ROS. (PDF) [file pbio.3002482.s011.pdf]

S1 Table

Enzyme family	Gene names	Annotated genes in *At* str. MWF001	Annotated genes in *Ct* str. MWF001
Superoxide dismutases	SodA, SodM	SodB (3)	SodB (2)
Catalases	KatA, KatE, KatG	KatG (1)	KatG (1)
Thiol peroxidase, thioredoxins	TpxD, TlpA, Etrx, TrxA, TrxB	TlpA (1), TrxA, TrxB (2), TrxC	TrxA (4), TrxC
Peroxiredoxin	AhpC, AhpD, Bcp	AhpC, AhpD, Bcp (2)	AhpC, AhpF, BcpB, Bcp
Glutathione reductase, glutaredoxin	gor, grxA	grxC, grxD	grxC, grxD

ROS resistance genes in *At* and *Ct*. We searched for a list of putative ROS-degrading enzymes from a recent review paper [57] by searching the annotated genes of our two strains. We show the gene families listed in [57], and whether we found genes of the same family in our two genomes with gene number shown in brackets. This analysis shows that gene presence/absence tells us little about which of the two strains is more resistant to ROS.
