# Supplementary material for: Oxidative stress changes interactions between 2 bacterial species from competitive to facilitative
Source: PLoS Biol. 2024 Feb 5;22(2):e3002482. doi: 10.1371/journal.pbio.3002482 (PMC10881020; doi:10.1371/journal.pbio.3002482)
Supplement: S2 Table — The compounds (LA, ROS, minimal medium nutrient) concentration unit is arbitrary (au). Code used for fitting is available at https://zenodo.org/records/10396269. (PDF) [file pbio.3002482.s012.pdf]

S2 Table

Symbol	Unit	Description	Model 1	Model 2
B	CFU.mL^{-1}	CFU concentration (*At* or *Ct*), proxy for bacterial population size		
C	au.mL^{-1}	LA concentration		
N	au.mL^{-1}	unknown MM nutrient concentration		
R	au.mL^{-1}	ROS concentration (proxy)		
r_{N1}	day^{-1}	maximum growth rate of *Ct* in MM	346.53	5.51
r_{N2}	day^{-1}	maximum growth rate of *At* in MM	891.43	126.46
r_{C1}	day^{-1}	maximum growth rate of *Ct* in LA	2.38	3.49
r_{C2}	day^{-1}	maximum growth rate of *At* in LA	1.94	2.37
K_{N1}	au.mL^{-1}	half-saturation constant (*Ct* in MM)	0.1	0.001
K_{N2}	au.mL^{-1}	half-saturation constant (*At* in MM)	1.01	0.36
K_{C1}	au.mL^{-1}	half-saturation constant (*Ct* in LA)	0.0006	0.04
K_{C2}	au.mL^{-1}	half-saturation constant (*At* in LA)	0.001	0.05
Y_{N1}	CFU.au^{-1}	yield (*Ct* in MM)	1.28×10^9	$4,08 \times 10^8$
Y_{N2}	CFU.au^{-1}	yield (*At* in MM)	3.14×10^8	3.27×10^8
Y_{C1}	CFU.au^{-1}	yield (*Ct* in LA)	1.38×10^9	5.79×10^9
Y_{C2}	CFU.au^{-1}	yield (*At* in LA)	2.383×10^9	10^{10}
β_1	mL.au^{-1}.day^{-1}	toxicity for *Ct* (LA in model 1, ROS in model 2)	0.3	8.58
β_2	mL.au^{-1}.day^{-1}	toxicity for *At* (LA in model 1, ROS in model 2)	3.36	20.03
γ_1	mL.au^{-1}.day^{-2}	toxicity accumulation of LA (model 1)	3.014×10^{-6}	
γ_2	mL.au^{-1}.day^{-2}	toxicity accumulation of LA (model 1)	0.563	
α_1	mL.CFU^{-1}.day^{-1}	rate of ROS detoxification by *Ct* (model 2)		4.6×10^{-6}
α_2	mL.CFU^{-1}.day^{-1}	rate of ROS detoxification by *At* (model 2)		0
d	day^{-1}	rate of LA oxidation (model 2)		0.11
l	day^{-1}	rate of ROS decay (model 2)		0.35
e	mL.au^{-1}.day^{-1}	ROS-triggered LA oxidation (model 2)		1.94
m	no unit	LA to ROS conversion factor (model 2)		0.88

Description of state variables and parameters used in the two models, with the estimates from fitting on mono-cultures. The compounds (LA, ROS, minimal medium nutrient) concentration unit is arbitrary (au).
