# Supplementary material for: Oxidative stress changes interactions between 2 bacterial species from competitive to facilitative
Source: PLoS Biol. 2024 Feb 5;22(2):e3002482. doi: 10.1371/journal.pbio.3002482 (PMC10881020; doi:10.1371/journal.pbio.3002482)
Supplement: S3 Table — We compute the squared errors (in log10) between the model ODE simulations and the data points to get a proxy for the goodness of the fit. We sum the errors for At and Ct growth at 0.1% and 0.75% LA to obtain a global goodness-of-fit proxy: for example, the goodness-of-fit of model 1 is 189.7. Two goodness-of-fit measures are considered: goodness-of-fit which is calculated using the mono-culture data and goodness-of-prediction which is calculated using the co-culture data. Model 1 and model 2 are the models presented in the main text (implicit toxicity and ROS-driven toxicity, respectively). Model 1 with no toxicity accumulation and model 2 with linear ROS dynamics are simpler versions of these models in which we ignore nontrivial dynamics such as the positive feedback loop in ROS generation (e = 0) and the accumulation of toxicity over time in model 1 (γ = 0). Model 1 has better goodness-of-fit measures for the mono-culture growth compared to model 2—this comparison must be taken with caution since model 2 also fits the ROS dynamics at the same time, and only uses 2 LA conditions where in model 1; 4 concentrations of LA were used to fit the mono-cultures. In terms of predicting the co-culture dynamics, model 2 with nonlinear ROS dynamics provides the most accurate prediction for the co-culture data. All code is available at https://zenodo.org/records/10396269. (PDF) [file pbio.3002482.s013.pdf]

S3 Table

Model	Mono-culture fit error	Co-culture prediction error
Model 1	189.7	483.0
Model 2	656.0	126.8
Model 1 (no toxicity accumulation, $\gamma = 0$)	258.43	916.8
Model 2 (linear ROS dynamics, $e = 0$)	693.6	272.9

Sum of squared-errors between the model and the data for the different models. We compute the squared errors (in $\log_{10}$) between the model ODE simulations and the data points to get a proxy for the goodness of the fit. We sum the errors for *At* and *Ct* growth at 0.1% and 0.75% LA to obtain a global goodness-of-fit proxy: for instance, the goodness-of-fit of model 1 is 189.7. Two goodness-of-fit measures are considered: goodness-of-fit which is calculated using the mono-culture data and goodness-of-prediction which is calculated using the co-culture data. Model 1 and model 2 are the models presented in the main text (implicit toxicity, and ROS-driven toxicity, respectively). Model 1 with no toxicity accumulation and model 2 with linear ROS dynamics are simpler versions of these models in which we ignore non-trivial dynamics such as the positive feedback loop in ROS generation ($e = 0$), and the accumulation of toxicity over time in model 1 ($\gamma = 0$). Model 1 has better goodness-of-fit measures for the mono-culture growth compared to model 2 - this comparison must be taken with caution since model 2 also fits the ROS dynamics at the same time, and only uses 2 LA conditions where in model 1, 4 concentrations of LA were used to fit the mono-cultures. In terms of predicting the co-culture dynamics, model 2 with non-linear ROS dynamics provides the most accurate prediction for the co-culture data.
