# Supplementary material for: Oxidative stress changes interactions between 2 bacterial species from competitive to facilitative
Source: PLoS Biol. 2024 Feb 5;22(2):e3002482. doi: 10.1371/journal.pbio.3002482 (PMC10881020; doi:10.1371/journal.pbio.3002482)
Supplement: Attachment — Submitted filename: Review- Oxidative stress changes interactions between two bacterial species (1).pdf [file pbio.3002482.s014.pdf]

Oxidative stress changes interactions between two bacterial species

In this manuscript, the authors explore the stress-gradient hypothesis (SGH) by assessing how different concentrations of linoleic acid (LA) affect the interaction between the bacteria *Agrobacterium tumefaciens* (*At*) and *Comamonas testosteroni* (*Ct*). They find that at low concentrations, both species can utilize LA as a carbon source, but at higher concentrations it becomes toxic to *At*. In addition, the authors demonstrate that, as expected in the framework of SGH, at low LA (low stress) concentrations the species compete, but at high concentrations (high stress) *Ct* facilitates the growth of *At.* Toxicity to *At* appears to be caused by the presence of ROS, which *Ct* can neutralize, thus improving the growth of *At* at these concentrations. When the antioxidant TBHQ is added to the high LA concentrations, and *At* no longer required the protection of *Ct*, the interaction is reverted to competition, similar to the lower LA concentrations.

The paper presents an interesting example of how interaction types are environment dependent. This case study is an interesting model for studying SGH as there is information regarding the mechanistic details underlying the interaction, which is based on detoxification, rather than the typical cross-feeding used in many other microbial model systems. This mechanistic understanding enables inferring the values of different parameters and offering insight into the dynamics of the interaction and their dependence on the environment. The generality of the findings awaits further studies (e.g. a gradient of concentrations, different compounds or species, longer timescales). However, these future directions do not diminish the value of this study.

<u>Main comments:</u>

1. It is not clear to what extent is Ct affected by competition for LA with At. In both the model and the experimental data *Ct* appears unaffected by the presence of *At*. As *At* grows well at both 0.01% and as well as 0.75% when TBHQ is added to the media, these results seem inconsistent with the statement *"showing that there was competition for LA"* (Line 95). If *Ct* is not affected by the presence of *At*, amensalism may be more accurate than competition.

2. More importantly, it's not clear why Ct is unaffected by the presence of At, even when At grows well. What could account for this lack of response by Ct? Could it be due to the fact that there is substantial growth even in the absence of an added carbon source? (as seen in Figure 1 and detailed in the model). Perhaps there is actually little niche overlap between Ct and At, with each consuming mostly a different resource when LA is abundant? While the authors may not have conclusive experimental data to answer these questions, the fitted model can reveal at list a plausible explanation.

3. It is worth discussing other effects TBHQ may have on the bacteria. For example, is it possible it may be used as a nutrient for *At*? Was the growth of the bacteria tested with TBHQ in the absence of LA (e.g. as a sole carbon source)? Lasty, in the methods there is no mention of how much TBHQ was added; besides the importance of recording this reproducibility purposes, it would give a sense of other effects it may potentially have on the bacteria.

4. Model 2 predicts that at 0.1% LA, *At* should go extinct after ~6 transfers (Fig. 4C). This is surprising given that the experimental data shows no signs of decline up to transfer 5 (Fig. 4B). It is unfortunate that the experiments could not be extended a bit further to test this model prediction, but it would be good to show simulated trajectories and check whether they are consistent with an abrupt drop in *At* density shortly after transfer 5. Furthermore, is there really no long-term (beyond 10 transfers) coexistence region in the model? How robust are these long-term coexistence results to the

uncertainty in the inferred model parameters?

5. I have several concerns regarding the mathematical modeling. First, some details regarding the modeling are lacking:

    a. The fit of the models is only shown for two LA concentrations. In particular, no data is shown for the fit in the absence of LA, used to infer the growth parameters on the unknown nutrient, and the fit to the ROS proxy in cell-free media, used to infer the parameters regarding ROS generation and spontaneous degradation.

    b. The objective function that was minimized during the fit is not stated explicitly. Is it least-squares on a linear or log scale? Were there any bounds imposed on parameter values? Was any complexity penalty imposed?

    c. No units are provided for the fitted parameter values.

    d. No goodness-of-fit measures are provided.

6. It is not clear to me to what extent these models are meant as "strategic models" that provide a simple potential mechanism explaining the data and should only fit the data qualitatively, or as "predictive models" that should fit the data quantitatively and be able to predict the systems behavior under new conditions/longer times. On the one hand, the language used in the manuscript and the lack of details regarding the goodness of fit suggest that it former, whereas the fact that the model was used to predict long-term coexistence suggest the latter. It would be helpful if the authors were more explicit on how the purpose of their model.

7. Since the models are used to make prediction regarding long-term coexistence, I am concerned that they include several non-trivial terms, such as the increase in toxicity with time of the first model, and the self-induced generation of ROS in the second model. It is hard to gauge to what extent these terms are warranted and are not overfitting the data. I am not necessarily advocating for performing rigorous model selection (e.g. via a likelihood ratio test, or cross-validation), but at least a visual comparison with the fit of simpler versions where these terms are absent should be provided.

8. The second model's superior fit is used to justify general claims regarding the necessity to explicitly model the ROS intermediate:

    "one resource and one inhibitor can allow two species to coexist, while a single compound can only do so under very restrictive conditions" (Lines 165, 166)

    "Within the CR framework, we found that whether we model a single compound whose effect changes or two compounds – one resource and one toxin – has a huge influence on coexistence" (Lines 280-282)

    It seems plausible that the improvement of accuracy is due to specific details of the models at hand, not solely their dimensionality. As mentioned above, the second model includes some non-trivial terms that may help it provide a better fit to the data. It seems likely that a more complex model where LA is directly toxic, for instance involving stronger non-linearities in the toxicity term, could be more accurate and also allow for a wider range of *At* survival. Therefore, I suggest tempering the

general statements mentioned above.

Minor Recommendations

I. Since ROS is generated even in the absence of cells, was the timing of media preparation before inoculation well-controlled in all experiments? If so, further details about the protocol are needed.

II. Generally, I found the presentation of the motivation and reasoning behind the first mathematical confusing while reading. I suggest being more explicit about the motivation for this model upfront and potentially foreshadowing its limitations.

III. It's not clear to what extent this work is intended to be relevant to the interaction between these species in MWFs. The statement (Lines 70-73) "*We selected ten compounds commonly found in MWF and tested their effect on At and Ct in monoculture at different concentrations (Fig. 1A). As Ct was facilitating At in MWF [36], we were looking for compounds on which Ct could grow but that would be challenging for At*" indicates that LA was selected since it could potentially capture the interaction observed in MWF. However, Citric acid appears to also be a good candidate, while other compounds (i.e. petroleum sulfonate) show the opposite trend. Moreover, it's not mentioned whether the concentrations tested here are comparable to concentrations of these compounds in MWF. Therefore, it is hard to gauge the relevance of this interaction mechanism in the complex MWF medium.

IV. *"who drives whom extinct and who promotes whose growth"* (Line 8)
These are presented as binary opposites, but that is not the case. There are options in between these, such as one species reducing another's growth, but not sufficiently to lead to the extinction of the affected species.

V. *"Modern Coexistence Theory [25, 26] focuses on species-species interactions with a general Lotka-Volterra framework"* (Lines 28-30)
Though MCT is not a mechanistic or resource-explicit framework, it is not limited to the generalized Lotka-Volterra framework. Moreover, many of the interesting coexistence mechanisms are explicitly based on the fact that interactions are non-linear.

VI.
"To distinguish At *and* Ct *when growing in the co-culture, bacteria were also plated on LB agar supplemented with 14.25g/ml of sulfamethoxazole and 355 0.75g/ml of trimethoprim to count only* At *colonies. Moreover, the GFP marker of* At *further helped to differentiate* At *and* Ct *colonies."* (Lines 354-357)
It is unclear then how a final number was arrived at. How was the information regarding CFUs on the non-selective media, CFUs on the selective media, and GFP signal combined? How stable is GFP during the experiment- is it on plasmid, or integrated into the genome?

VII. *"These results also showed that toxicity was not caused by the increase of LA concentration itself, but rather by the accumulation of ROS."* (Lines. 125-126)
Though these results are consistent with this hypothesis, they do not provide direct evidence for it.

VIII. *"Fig. 1A shows that different compounds can be toxic in a species- and concentration-dependent way, suggesting that there is nothing particular about the compound*

we chose to study." (Lines 218-220)
Different sensitivities alone are not sufficient for facilitation, which also requires detoxification of the toxic compound (or another mechanism lowering the sensitivity of the affected species). Is such detoxification common? It would be useful to be more explicit about this point.

IX. *"is ROS being taken up and neutralized intracellularly or is Ct secreting extracellular enzymes that eliminate ROS?"* (Lines 229-230)
It's worth suggesting ways of tackling this interesting question. For example, experiments where At is grown on a mixture of fresh and spent media from a Ct culture may help distinguish these options.

X. P-values on Fig. 1 are missing a 0 after the decimal point (0.5 instead of 0.05 etc).

XI. In Fig. 1A it seems like the AUC of *At* at the highest concentration of LA is similar to the AUC of the control. This is inconsistent with what is shown in panel B.

XII. In Fig. 3B, I found it confusing that the cell free and coculture data are shared across the experimental panels and colored according to the species. In addition, comparing these data with the model is d ifficult - it is hard to locate the cell-free ROS curve in the model.

XIII. Ref 13 (Line 18) does not mention siderophores production.

XIV. Some references are not properly formatted (e.g. 27, 35, 38)

XV. Line 399: 0.075% is written instead of 0.75%
