# Supplementary material for: Oxidative stress changes interactions between 2 bacterial species from competitive to facilitative
Source: PLoS Biol. 2024 Feb 5;22(2):e3002482. doi: 10.1371/journal.pbio.3002482 (PMC10881020; doi:10.1371/journal.pbio.3002482)
Supplement: Attachment — Submitted filename: DiMartino_Picot_responseToReviewers.pdf [file pbio.3002482.s015.pdf]

Response to referees
Manuscript number: PBIOLOGY-D-23-01367R1
Title: "Oxidative stress changes interactions between two bacterial species"

We are very grateful to all three reviewers for their comments, which we think have greatly helped to clarify and improve the manuscript. Reviewers' comments are written in black below, and our responses in blue font and indented text. Direct changes to the text are underlined here and highlighted in blue in the revised manuscript. We have since also uploaded the complete genomes of the species used in this study to NCBI and are including the reference to that in the methods section.

Reviewer #1:

Understanding how species interactions depend upon the environment is a central question in ecology. One pattern that has been observed is that species interactions often become more positive when the environment is more challenging for the species (the so-called stress gradient hypothesis (SGH)). Here the authors study a co-culture of *Agrobacterium tumefaciens* (henceforth At) and *Comamonas testosteroni* (Ct) growing in a defined medium containing linoleic acid (LA) as the sole carbon source. The authors find that this carbon source becomes toxic for At at high concentration due to the accumulation of Reactive Oxygen Species (ROS). Using a combination of experiment and modeling, the authors explain how the interactions in the two-species co-culture move towards more competition (or facilitation) by reducing (or increasing) the initial LA concentration, consistent with expectations from the SGH. In addition, they find that adding an antioxidant reduces
environmental toxicity, thus moving the interaction back to competition. I very much enjoyed the clear writing and the elucidation of a mechanistic origin behind how the environment can determine the sign of interactions between species, and I have only one question to be addressed (I will also note that Sara Mitri's papers are unusually carefully edited, as a typical submitted paper contains a dozen typos, whereas I didn't notice any in this paper nor in some other papers of hers that I have reviewed…. Thanks to the authors for putting in this extra effort).

> We thank the reviewer for the positive feedback and are very glad he sees the value of our work. Indeed, we put a lot of effort into writing clearly and cleanly and are pleased that it is being appreciated!

Long-term dynamics (line 194): "We found no conditions where the long-term stable coexistence of the two species was possible." It seems to me that At in the co-culture experiments is surviving for a pretty long time, and there is no evident decrease in the cell density over five cycles. I would have interpreted this as reasonable evidence for "long-term" survival, so from that standpoint I would say that Model 2 is still not consistent with the experimental data. (Of course, the Ct population density dips on the final cycle, and this could have unpredictable effects for the co-culture…. However, this is probably just due to experimental variation on that cycle?). Do the authors really think that there co-culture would not survive indefinitely? I suspect that there are some experimental conditions where they would… In particular, cross-feeding is such a ubiquitous phenomenon that most single carbon sources can support dozens of species / strains. Obviously, if you write down

a resource explicit model that doesn't include cross-feeding then you will predict that only a single species will survive, but that is because the model has assumed that there is no cross-feeding. The model that the authors develop is clearly capturing some essential dynamics that are present within the community, but it is still making a strong assumption about the lack of cross-feeding, and without clear evidence of competitive exclusion at high resource concentration it seems odd to end the paper by making a strong modeling prediction that may or may not be justified.

We agree that these statements could be much clearer. What we meant to say was that the model finds no conditions under which the two species can stably coexist. It's true, though, that in the experiments coexistence looks much more likely at least in the short-term. We also suspect that there is cross-feeding between the two species. We actually spent around a year chasing this hypothesis with mass spec experiments (the collaborators are mentioned in the acknowledgements). Unfortunately, we never managed to get clean measurements out of this approach (our controls never showed what one would expect from them), possibly because of the presence of ROS. We finally decided to abandon this angle. It's true, however, that we do not explain that cross-feeding is absent from the current model and it therefore may be underestimating the potential for coexistence.

Accordingly, we have expanded our single sentence in the discussion into the following paragraph: "One indication that our model may be underestimating the potential for coexistence, is that in Fig. 4B, *At* does not appear to be close to extinction in either condition at transfer 5, while our model predicts that it should go extinct between transfers 6 and 10 (S3 Fig). This discrepancy leads us to suspect that despite our efforts to quantitatively predict outcomes of these co-cultures, our model is likely still missing further interaction layers between the two species like cross-feeding that were not observed in this study. Detecting cross-feeding in our system was challenging, as we found LA to not be easily amenable to standard chemical analysis methods. Understanding the coupling between detoxification and cross-feeding, and their effect on long-term coexistence is left for future work."

Reviewer #2:

In this manuscript, the authors explore the stress-gradient hypothesis (SGH) by assessing how different concentrations of linoleic acid (LA) affect the interaction between the bacteria *Agrobacterium tumefaciens* (At) and *Comamonas testosteroni* (Ct). They find that at low concentrations, both species can utilize LA as a carbon source, but at higher concentrations it becomes toxic to At. In addition, the authors demonstrate that, as expected in the framework of SGH, at low LA (low stress) concentrations the species compete, but at high concentrations (high stress) Ct facilitates the growth of At. Toxicity to At appears to be caused by the presence of ROS, which Ct can neutralize, thus improving the growth of At at these concentrations. When the antioxidant TBHQ is added to the high LA concentrations, and At no longer required the protection of Ct, the interaction is reverted to competition, similar to the lower LA concentrations.

The paper presents an interesting example of how interaction types are environment dependent. This case study is an interesting model for studying SGH as there is information regarding the mechanistic details underlying the interaction, which is based on detoxification, rather than the typical cross-feeding used in many other microbial model systems. This mechanistic understanding

enables inferring the values of different parameters and offering insight into the dynamics of the interaction and their dependence on the environment. The generality of the findings awaits further studies (e.g. a gradient of concentrations, different compounds or species, longer timescales). However, these future directions do not diminish the value of this study.

> We are glad the reviewer finds our work interesting and appreciates our effort to build a mechanistic understanding. We are currently working on entirely different projects to test the generality of our findings.

Main comments:

1. It is not clear to what extent is Ct affected by competition for LA with At. In both the model and the experimental data Ct appears unaffected by the presence of At. As At grows well at both 0.01% and as well as 0.75% when TBHQ is added to the media, these results seem inconsistent with the statement “showing that there was competition for LA” (Line 95). If Ct is not affected by the presence of At, amensalism may be more accurate than competition.

> It’s true that our statement was not very accurate. We now changed it to “showing that there was competition for LA that negatively affected the growth of At.” (as we think that there is still competition for the sole resource, just that Ct is able to take it up faster). And two sentences later we added: “Taken together, we classify the interactions between the two species as “ammensalism” at 0.1% LA and “commensalism” at 0.75% LA, with only one species being negatively or positively affected by the other, respectively [Mitri & Foster, 2013].” In a few places in the text, we also specified that there was competition or facilitation “from *Ct* to *At*”, rather than “between *At* and *Ct*”.

2. More importantly, it’s not clear why Ct is unaffected by the presence of At, even when At grows well. What could account for this lack of response by Ct? Could it be due to the fact that there is substantial growth even in the absence of an added carbon source? (as seen in Figure 1 and detailed in the model). Perhaps there is actually little niche overlap between Ct and At, with each consuming mostly a different resource when LA is abundant? While the authors may not have conclusive experimental data to answer these questions, the fitted model can reveal at list a plausible explanation.

> We think that *Ct* can simply take up LA faster. LA is the only carbon source we add to the medium, although we detected quite a bit of growth without LA suggesting that there are traces of carbon in the base medium. We now include a new supplementary figure (new S1 Fig) showing how much each of the species grows in the base medium without LA. There is still a small competitive effect of *At* on *Ct* in the base medium, but the effect on *At* only appears once we add in LA. The growth parameters on the MM base medium are included in the model (new S8 Fig).

3. It is worth discussing other effects TBHQ may have on the bacteria. For example, is it possible it may be used as a nutrient for At? Was the growth of the bacteria tested with TBHQ in the absence of LA (e.g. as a sole carbon source)? Lasty, in the methods there is no mention of how much TBHQ was added; besides the importance of recording this reproducibility purposes, it would give a sense of other effects it may potentially have on the bacteria.

This is a very good point and one that we had already checked. We now include a new supplementary figure (new S2 Fig) showing that the bacteria are neither positively nor negatively affected by TBHQ at the concentration we chose. We also explain this more clearly in the revised text.

Thank you also for noticing that we had forgotten to write a methods section about TBHQ! We have now added that section explaining how it was prepared and how we decided on the concentration used.

4. Model 2 predicts that at 0.1% LA, At should go extinct after ~6 transfers (Fig. 4C). This is surprising given that the experimental data shows no signs of decline up to transfer 5 (Fig. 4B). It is unfortunate that the experiments could not be extended a bit further to test this model prediction, but it would be good to show simulated trajectories and check whether they are consistent with an abrupt drop in At density shortly after transfer 5. Furthermore, is there really no long-term (beyond 10 transfers) coexistence region in the model? How robust are these long-term coexistence results to the uncertainty in the inferred model parameters?

It is indeed unfortunate that our data does not extend beyond 5 transfers. The 5th transfer coincided with the first lock-down during the pandemic. In any case we agree that coexistence seems to be more likely in the experiments, and our hypothesis is that there may be cross-feeding between the two species that we could not detect and are therefore not including in the model (see response to reviewer #1). In other words, in the absence of cross-feeding (in the model) we expect no long-term coexistence. In reality, there may be more, but we unfortunately do not have the data to test it.

Regarding the model, we now include a new supplementary figure (new S3 Fig) where we show the trajectories in the model, which are not abrupt. We have also expanded this point in the discussion (response to reviewer #1).

Finally, the fact that we find no region of coexistence in the model regardless of the model parameters is unsurprising based on previous work, as the underlying competition for LA eventually drives the weaker grower extinct. In the absence of cross-feeding, this is inevitable in the vast majority of the parameter space (Stewart & Levin 1973).

5. I have several concerns regarding the mathematical modeling. First, some details regarding the modeling are lacking:
a. The fit of the models is only shown for two LA concentrations. In particular, no data is shown for the fit in the absence of LA, used to infer the growth parameters on the unknown nutrient, and the fit to the ROS proxy in cell-free media, used to infer the parameters regarding ROS generation and spontaneous degradation.

Thank you for noticing. We have now added two supplementary figures: S8 Fig for the fit in the minimal medium and S7 Fig for the ROS generation and spontaneous degradation in cell-free media.

b. The objective function that was minimized during the fit is not stated explicitly. Is it least-squares on a linear or log scale? Were there any bounds imposed on parameter values? Was any complexity penalty imposed?

> We have now added these details to the methods section "All parameter estimations were obtained using the modFit function from FME package (version 1.3.6.1) in R version 4.1.0, which uses the Levenberg-Marquardt algorithm from the nls.lm function (min.pack package). The objective function was defined by the log10 of the error between data points and the fit (both for fitting CFU data or ROS data). Initial parameter sets were obtained by fitting the data by trial and error. Parameter values were explored linearly or on a log scale, which led to similar estimates. In the linear exploration an upper bound was set to 10^10 and a lower bound to 0. In the main text, we chose one arbitrary parameter set for each model, which are not intended as being the absolute best-fit, but more as a representative of the output from the fitting routine."

c. No units are provided for the fitted parameter values.

> We have added a table with the parameter description units and values in the Supplement (S2 Table).

d. No goodness-of-fit measures are provided.

> We have added these in S3 Table and discuss this point in the method section:
> In S3 Table, we present some quantitative measures for the performance of the models. In particular, the goodness-of-prediction measure allows us to compare the prediction ability of the model by computing the error between the co-culture growth predicted by fitting the mono-culture data, and the actual co-culture data. The goodness-of-fit measure allows us to compare different versions of model 1 or of model 2 to assess whether to add complexity to the model. The goodness-of-fit measures of model 1 and model 2 are not directly comparable firstly because the mono-culture fitting routine in model 1 uses 4 concentrations of LA, while the fitting of model 2 uses only two; secondly, in model 2, the ROS dynamics are included in the fitting routine, which may explain why the overall goodness-of-fit (based on the CFU data) is better in model 1 than in model 2.

6. It is not clear to me to what extent these models are meant as "strategic models" that provide a simple potential mechanism explaining the data and should only fit the data qualitatively, or as "predictive models" that should fit the data quantitatively and be able to predict the systems behavior under new conditions/longer times. On the one hand, the language used in the manuscript and the lack of details regarding the goodness of fit suggest that it former, whereas the fact that the model was used to predict long-term coexistence suggest the latter. It would be helpful if the authors were more explicit on how the purpose of their model.

> This is a good question. While we think it may be very difficult for our model to be comprehensive enough to capture all the necessary elements to make accurate predictions, we still see it as a predictive model, as we assess its performance in predicting co-culture dynamics based on mono-culture fitting. However, we agree that in the end it's unclear how

predictive the model really is, especially regarding long-term coexistence. We therefore added the paragraph mentioned in our response to point 4 above and a small sentence to the following paragraph on our lessons for building such models: "and while we suspect that our model is still missing some of these factors to make it predictive in the long-term, it nevertheless served to understand our system, how to control it, and to generate the testable hypothesis that toxicity may extend the duration of coexistence".

7. Since the models are used to make prediction regarding long-term coexistence, I am concerned that they include several non-trivial terms, such as the increase in toxicity with time of the first model, and the self-induced generation of ROS in the second model. It is hard to gauge to what extent these terms are warranted and are not overfitting the data. I am not necessarily advocating for performing rigorous model selection (e.g. via a likelihood ratio test, or cross-validation), but at least a visual comparison with the fit of simpler versions where these terms are absent should be provided.

This is also a good point. Prior to submission, we had discussed at length whether we could use a model selection approach. We finally decided that this did not make sense because the data to infer the parameters is not the same in the two models (CFU only or ROS + CFU). We instead included a measure of final sum-of-squares error to have some quantification (S3 Table). We discuss these points in the caption of S3 Table: "We compute the squared errors (in log10) between the model ODE simulations and the data points to get a proxy for the goodness of the fit. We sum the errors for At and Ct growth at 0.1% and 0.75% LA to obtain a global goodness-of-fit proxy. Two goodness-of-fit measures are considered: goodness-of-fit which is done on the mono-cultures and goodness-of-prediction which is done on the co-cultures. Model 1 and model 2 are the models presented in the main text (implicit toxicity, or ROS-driven toxicity). Model 1 with no toxicity accumulation and model 2 with linear ROS dynamics are simpler versions of these models in which we ignore non-trivial dynamics such as the positive feedback loop in ROS generation, and the accumulation of toxicity over time in model 1. Model 1 has better goodness-of-fit measures for the mono-culture growth compared to model 2 - this comparison must be taken with caution since model 2 also fits the ROS dynamics at the same time, and only uses 2 LA conditions where in model 1, 4 concentrations of LA were used to fit the mono-cultures. In terms of prediction of the co-culture dynamics, model 2 with non-linear ROS dynamics provides the most accurate prediction for the co-culture growth." as well as in the method section (see response to 5d).

We also liked the idea of adding a visual comparison, though, so we have added two supplementary figures with the fit of simpler versions of the model: S6 where the accumulation of toxicity over time is absent (gamma=0), and S9 in which there is no self-induced generation of ROS in the model (e=0).

8. The second model's superior fit is used to justify general claims regarding the necessity to explicitly model the ROS intermediate:
"one resource and one inhibitor can allow two species to coexist, while a single compound can only do so under very restrictive conditions" (Lines 165, 166)
"Within the CR framework, we found that whether we model a single compound whose effect changes or two compounds – one resource and one toxin – has a huge influence on coexistence" (Lines 280-282)

It seems plausible that the improvement of accuracy is due to specific details of the models at hand, not solely their dimensionality. As mentioned above, the second model includes some non-trivial terms that may help it provide a better fit to the data. It seems likely that a more complex model where LA is directly toxic, for instance involving stronger non-linearities in the toxicity term, could be more accurate and also allow for a wider range of At survival. Therefore, I suggest tempering the general statements mentioned above.

> The statements we initially had in the paper were based on parameter sweeps and quite extensive efforts to get the first model to fit the data well. Through these parameter sweeps we also found that getting coexistence between the two species was very challenging. Once we changed to the new model, things fell into place quite easily, and the coexistence region was large under quite a large parameter range. At that point, we had also worked out the mechanism experimentally and it would be odd not to update our model based on our experimental understanding of the system. We also stress that beyond coexistence, the main argument for the superiority of model 2 compared to model 1 is based on its predictive ability for the co-culture dynamics. If model 2 was better than model 1 in fitting the mono-cultures but would not do better in predicting co-cultures, we would agree that it might only be a question of overfitting. But here what is interesting is that including the ROS-driven toxicity better predicts the co-culture dynamics so we think that adding more mechanistic information does improve the understanding of the interaction between our two species.
>
> That said, the reviewer is correct in pointing out that just because we didn't find conditions under which the first model gives us coexistence, doesn't mean it's not possible. We have therefore modified the statements to tone them down somewhat: "we know from early theoretical work that one resource and one inhibitor are predicted to allow two species to coexist, while a single compound should only do so under very restrictive conditions" and "whether we model a single compound whose effect changes or two compounds - one resource and one toxin - likely increases the parameter space under which the two species coexist".

Minor Recommendations

I. Since ROS is generated even in the absence of cells, was the timing of media preparation before inoculation well-controlled in all experiments? If so, further details about the protocol are needed.

> Yes, ROS is generated in the absence of cells, as shown in Fig. 3B (cell-free). We have added a few words on first mention of ROS to make this clearer: "Based on the literature, we hypothesized that spontaneous oxidation of LA might release reactive oxygen species (ROS) [44–47] even in the absence of bacteria." And yes, we always prepared the media in the same way. We have added a sentence to the methods section: "All media were prepared fresh on the morning of each experiment, as we were aware that these compounds, particularly LA could oxidize over time."

II. Generally, I found the presentation of the motivation and reasoning behind the first mathematical confusing while reading. I suggest being more explicit about the motivation for this model upfront and potentially foreshadowing its limitations.

We were unsure why our text was confusing. When we introduce the model we say “To generate quantitative predictions on the behavior of these two species in mono- and co-culture, we developed a mathematical model” and later we were quite explicit about why our model failed to be predictive and the need for an updated model: “Although these results matched the model predictions qualitatively, At's growth in co-culture was greatly underestimated by the model (Fig. 2B, D, green dashed lines). Furthermore, using the parameters estimated from all mono-cultures, the model does not correctly predict the hump-shaped growth of At at 0.75% LA (Fig. 2D), even though it already assumes the accumulation of toxicity. This suggests that estimating model parameters where both growth and death are caused by a single compound is challenging.” We also now discuss its limitations in more depth in the discussion section.

III. It’s not clear to what extent this work is intended to be relevant to the interaction between these species in MWFs. The statement (Lines 70-73) “We selected ten compounds commonly found in MWF and tested their effect on At and Ct in monoculture at different concentrations (Fig. 1A). As Ct was facilitating At in MWF [36], we were looking for compounds on which Ct could grow but that would be challenging for At” indicates that LA was selected since it could potentially capture the interaction observed in MWF. However, Citric acid appears to also be a good candidate, while other compounds (i.e. petroleum sulfonate) show the opposite trend. Moreover, it’s not mentioned whether the concentrations tested here are comparable to concentrations of these compounds in MWF. Therefore, it is hard to gauge the relevance of this interaction mechanism in the complex MWF medium.

We appreciate that our intentions were not very explicit. Our goal is rather to understand the general ecological principles than to explain what was happening in the MWF, which is clearly a much more complex environment. We do agree, though, that for a reader keen to understand interactions in MWF, it would at least be helpful to note whether these concentrations are expected to be similar and how we would expect other ingredients to affect the interactions, as they may go in opposite directions. We have added more details to the methods section justifying our choice: “Previous studies show that bacteria are susceptible to benzotriazole and formaldehyde [Aarestrup 2004, Wu 1998], so we chose concentrations above and below these thresholds. Petroleum sulfonate and naphthenic petroleum oil concentrations were chosen to resemble those commonly found in MWF [Byers 2017]. Monoethanolamine, triethanolamine, citric acid and morpholine concentrations were chosen to be similar to those already tested by the developers of the consortium [van der Gast 2002]”.

We also explicitly address this point in the results section: “Our goal here is not to explain what we observed in MWF, but rather to design a comparable model chemical environment in which to study interactions in a more controlled way”.

IV. “who drives whom extinct and who promotes whose growth” (Line 8)

These are presented as binary opposites, but that is not the case. There are options in between these, such as one species reducing another's growth, but not sufficiently to lead to the extinction of the affected species.

Good point, we have changed it to be less binary: "who restricts or enhances whose growth"

V. "Modern Coexistence Theory [25, 26] focuses on species-species interactions with a general Lotka-Volterra framework" (Lines 28-30)
Though MCT is not a mechanistic or resource-explicit framework, it is not limited to the generalized Lotka-Volterra framework. Moreover, many of the interesting coexistence mechanisms are explicitly based on the fact that interactions are non-linear.

We agree that this summary of MCT may be a bit brief, but we found no reason to go into great depth here. Our intention was more to emphasize that we are moving to more resource-explicit models. We have now changed it to "Modern Coexistence Theory [25, 26] focuses on species-species interactions often with a general Lotka-Volterra framework"

VI. "To distinguish At and Ct when growing in the co-culture, bacteria were also plated on LB agar supplemented with 14.25g/ml of sulfamethoxazole and 0.75g/ml of trimethoprim to count only At colonies. Moreover, the GFP marker of At further helped to differentiate At and Ct colonies." (Lines 354-357).
It is unclear then how a final number was arrived at. How was the information regarding CFUs on the non-selective media, CFUs on the selective media, and GFP signal combined? How stable is GFP during the experiment- is it on plasmid, or integrated into the genome?

Ct colonies always grew on non-selective media after 1 day, which is when we counted them. At this point, no At colonies would have appeared. We counted At on the selective media and used the GFP signal whenever there was doubt that colonies were in fact At. This was rare. The GFP is integrated into the genome and the resistance to SXT is native to At, not engineered. We now clarify this and refer to a previous publication, where this is described in more detail.

VII. "These results also showed that toxicity was not caused by the increase of LA concentration itself, but rather by the accumulation of ROS." (Lines. 125-126)
Though these results are consistent with this hypothesis, they do not provide direct evidence for it.

We have toned this down to: "These results are in line with the idea that toxicity was not caused by the increase of LA concentration itself, but rather by the accumulation of ROS."

VIII. "Fig. 1A shows that different compounds can be toxic in a species- and concentration-dependent way, suggesting that there is nothing particular about the compound we chose to study." (Lines 218-220)
Different sensitivities alone are not sufficient for facilitation, which also requires detoxification of the toxic compound (or another mechanism lowering the sensitivity of the affected species). Is such detoxification common? It would be useful to be more explicit about this point.

Thank you for noticing that detoxification is also a requirement. We have changed the sentence to: “Fig. 1A shows that different compounds can be toxic in a species- and concentration-dependent way, and if species are also able to remove their toxic effect they should behave similarly to the compound we chose to study.”

IX. "is ROS being taken up and neutralized intracellularly or is Ct secreting extracellular enzymes that eliminate ROS?" (Lines 229-230)
It’s worth suggesting ways of tackling this interesting question. For example, experiments where At is grown on a mixture of fresh and spent media from a Ct culture may help distinguish these options.

Spent medium experiments are difficult to perform in this medium as LA does not dissolve and so gets stuck in filters. However, one could disentangle this effect with a simpler source of ROS, such as hydrogen peroxide. We have added this sentence to the end of that paragraph: “Spent-media experiments in a medium containing a simpler carbon source in addition to a ROS could help to distinguish intra- and extracellular ROS elimination by *Ct*.”

X. P-values on Fig. 1 are missing a 0 after the decimal point (0.5 instead of 0.05 etc).

Thanks for noticing, we have fixed this.

XI. In Fig. 1A it seems like the AUC of At at the highest concentration of LA is similar to the AUC of the control. This is inconsistent with what is shown in panel B.

The reason why they appear to be similar is because the comparison is done using the AUC, which amplifies the effect of large population sizes at the beginning of the curve. Basically, 10^6 + 10^6 + 10^6 with some noise on the order of 10^5 (we are counting colonies at that dilution), is not significantly different from 10^6 + 10^3 + 0 again with noise on the order of 10^5. These are not real data or a correct AUC calculation, but an illustrative example.

XII. In Fig. 3B, I found it confusing that the cell free and coculture data are shared across the experimental panels and colored according to the species. In addition, comparing these data with the model is difficult - it is hard to locate the cell-free ROS curve in the model.

We like the suggestion and have changed its color to black. We also now highlight that in the model the line is below the At mono-culture line in the figure caption.

XIII. Ref 13 (Line 18) does not mention siderophores production.

We were referencing it because of the cross-feeding, but it’s true that it doesn’t talk about siderophores, so we have now also added another citation.

XIV. Some references are not properly formatted (e.g. 27, 35, 38)

Thanks, we have fixed these.

XV. Line 399: 0.075% is written instead of 0.75%

Thank you for noticing and going through the manuscript so meticulously!

Reviewer #3:

This study builds up on previous work by Dr. Mitri's group, studying how the ecology of microbial communities changes in response to harsher versus more benign environments. Here, they provide a detailed mechanistic characterization of the SGH hypothesis in their system, quantitatively establishing how the concentration of a single compound can modulate the sign of the ecological interaction through its associated toxicity. I find it quite brave to work with such a difficult system, and being able to disentangle it mechanistically has a lot of merit. It is also a beautiful example of how the back and forth between models and experiments can help mechanistically characterize the observed interactions and population dynamics. I also greatly appreciated the depth and breadth of the discussion, including the generalizability of the phenomenon, the multi-layered nature of even apparently simple microbial interactions, and the level of mechanistic detail that we need to include in our models. The findings are novel, the paper is very well written, the questions are answered rigorously and the limitations are discussed transparently. Thus, I am overall very enthusiastic with this contribution whose importance spans from community ecology in general to microbial ecology and microbiology.

We thank the reviewer for appreciating our work and seeing its merits! We genuinely hope future readers will also find it interesting!

Minor points

I can't help commenting on the growth with no added carbon source in Fig. 1. I acknowledge the authors transparency and care in documenting it with references. We have also noticed a similar phenomenon in our experiments, but it remains striking to me. Do authors know if perhaps these species are forming any storage molecule during "feast" times, e.g. during the precultures in TSB, that they are then using during the "famine" period?

We actually spent a significant amount of time trying to understand this point, as it made everything more complicated, including the model as we had to include this residual growth in our equations.

Our initial hypothesis was that cells were indeed storing molecules from previous growth in a rich medium to grow later in the MM. To test this hypothesis, we incubated each species in monoculture in MM at a high initial population size (10^7-10^8 CFU/ml) for either 24 or 48 hours, and then diluted them a 100-fold into fresh MM. At grew slightly worse after dilution, but Ct grew as well as before in both cases (see figure just below). It is still possible that 48 hours is not enough and we should do several dilutions in MM before rejecting this hypothesis, but we decided to move on and test others.

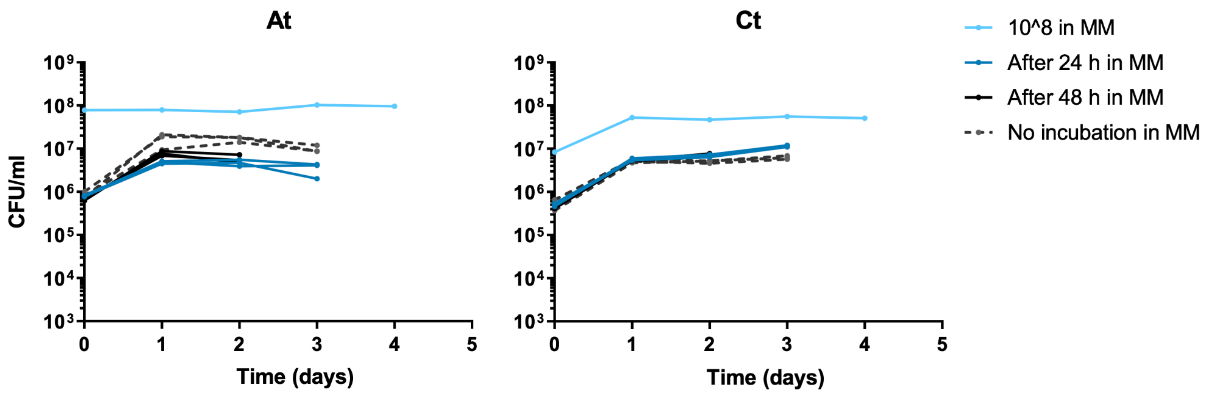

Another idea was that the small amounts of carbon in the MM could be enough for cells to grow on (Table 1 in the main text). We tested their growth in the regular Minimal Medium (MM), a variant of MM without EDTA and NTA as they were the only carbon-containing molecules (MM-), and a medium with only salts and phosphate (M9), which was the basis of the MM. We incubated the two species in monocultures in these three media at two different starting population sizes (10^5-10^6 and 10^3-10^4 CFU/ml) and found similar growth to the MM in almost all cases (see figure below).

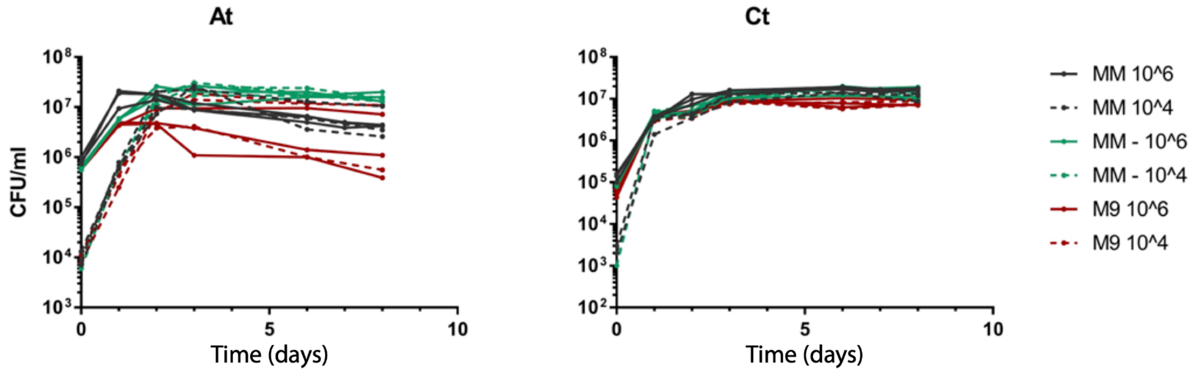

Suspecting that the water source might be contaminated, we also compared media prepared with ROTIPURAN®Ultra water (Roth), an extra pure water used for sample preparation in trace analyses to our standard ddH$_2$O. We also compared the glass tubes we use in this study with 14 ml Falcon® plastic tubes thinking that the glass tubes might contain residual carbon accumulated during the washing of the glassware. For these tests we used only At but found that it grew in all cases (figure below, 104 and 106 indicate starting population sizes of 10^4 and 10^6).

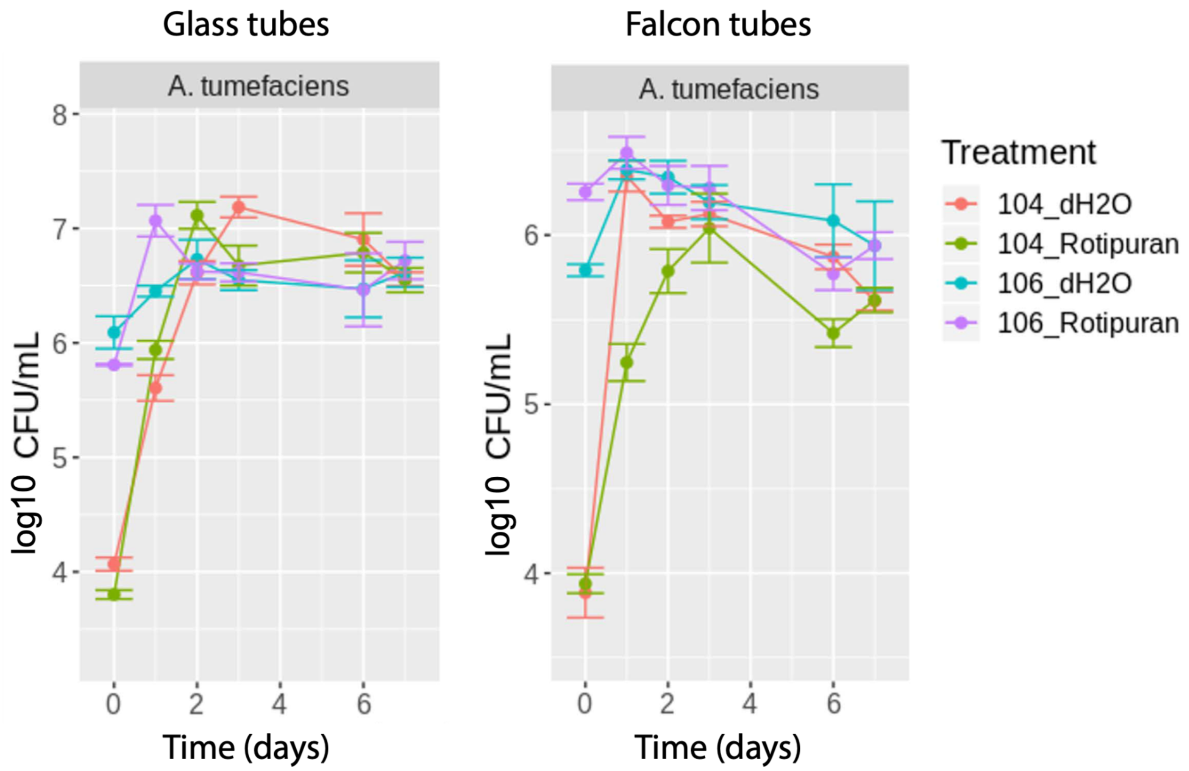

At this point, we still have some hypotheses that we have not tested: perhaps measuring population sizes using CFU/ml is biasing our results (if cells are clumped at the start, resulting in fewer colonies), or all our media powders are contaminated with some trace amounts of carbon. However, given that this was not the focus of the paper, we decided to put this issue aside, especially since we heard from others that we are not the only ones to observe this (hence the citations). We also decided not to include these data in the supplement, as the experiments were inconclusive and again, it seems to be a somewhat common phenomenon.

Statistical analysis is adequate throughout the paper. However, please clarify the statistical analysis reported in caption of Fig 1. If I understand correctly, in each case the t test was performed between a condition (compound X added at concentration Y, three replicates) and all the replicates of the condition with no added compound (how many?). Please report the N as well.

Thank you for noticing, we have added the information to the caption.

L355 - Could you please clarify how these compounds added to LB help distinguish the two species, or provide reference.

Our apologies, this seemed to have also confused reviewer #2. We now clarify this and refer to a previous publication, where this is described in more detail.
